# Using Queer Theory to Advance Understandings of ‘Queer Caregiving’ With LGBTQ+ Older Adults

**DOI:** 10.1111/scs.70178

**Published:** 2026-01-06

**Authors:** Steven Hall, Austin Oswald

**Affiliations:** ^1^ Faculty of Nursing University of Alberta Edmonton Alberta Canada; ^2^ School of Health and Human Performance Dalhousie University Halifax Nova Scotia Canada

**Keywords:** caregivers, LGBTQ+, lived experiences, older adults, queer theory

## Abstract

**Aims:**

This paper explores queer theory, its historical evolution, and its applications to the caregiving experiences of LGBTQ+ individuals caring for older adults. LGBTQ+ older adults often rely on informal caregiving from within their community. In this work, queer theory is employed as a critical theoretical lens to deconstruct societal norms and expectations that marginalise LGBTQ+ informal caregivers, referred to in this work as ‘queer caregivers’, emphasising the fluidity of identity and the non‐normative narratives of LGBTQ+ lives.

**Methods:**

Health systems must adopt more inclusive and culturally competent approaches to better support queer caregivers and their care recipients, which aligns with queer theory's challenge to normative structures. We propose the integration of queer theory into healthcare practices that work to recognise informal caregivers as a transformative pathway towards inclusivity, ensuring equitable care for all, regardless of sexual orientation or gender identity. We also provide recommendations for future research, such as exploring the intersectionality of queer caregivers and developing tailored support interventions.

**Conclusions:**

Embracing queer theory as a guiding framework in caregiver research, practice and policy will not only enhance understanding of queer caregiving but also contribute to the advancement of a more equitable and compassionate society that values and supports all caregivers, regardless of their sexual orientation or gender identity.

## 1 Introduction

The aging population is growing rapidly, with significant implications for social and healthcare services [1]. Within this demographic shift, lesbian, gay, bisexual, transgender and queer (LGBTQ+, with + denoting the range of sexual orientations and gender identities not explicitly mentioned in the acronym) older adults experience multiple vulnerabilities due to the structural marginalisation and inequities within social and health systems [2, 3]. Research highlights that LGBTQ+ older adults face distinct health issues due to a combination of factors and stress associated with their sexual minority status [4, 5]. LGBTQ+ older adults often bear the scars of historic and contemporary discrimination, impacting their well‐being and access to appropriate care [6]. They are more likely to experience physical, psychological and cognitive health conditions compared to the general population, a disparity that is compounded by substandard care and, at times, the outright denial of care by discriminatory social and healthcare providers [5, 7, 8, 9]. This body of evidence on LGBTQ+ older adults continues to grow in the emerging field of study known as ‘queer gerontology’ [4].

Many LGBTQ+ older adults also act as informal caregivers to their families of origin and families of choice [10, 11]. Families of origin are the families that LGBTQ+ individuals are born into or raised by, often a biological family; whereas, a family of choice (or ‘chosen family’) is a group of people an individual selects to form their own support system based on mutual love, respect and emotional connection [12]. In the broader societal context, informal caregivers (herein referred to as ‘caregivers’) provide essential health and social support to older adults, but they themselves are at risk of negative health outcomes, especially if they lack adequate support, posing a significant challenge for health systems [13, 14, 15]. Theory, in a broad sense, is essential for addressing health systems challenges because it provides structure, clarity and actionable frameworks for understanding and solving complex problems [16, 17]. When used appropriately, theory can guide intervention implementation in practice, inform decision‐making and policy formation, and address how inequalities and systemic factors impact health by guiding actions to reduce disparities [16, 17, 18].

It is critical to recognise that LGBTQ+ older adults are not a homogenous group, and their experiences of aging are shaped by the intersecting dynamics of gender, sexuality, race, class, ability and other identities. Research has shown that aging experiences differ significantly across subgroups under the LGBTQ+ ‘rainbow’, with each facing distinct historical and social pressures that influence how they navigate aging, healthcare and informal caregiving structures [19]. Intersections with race and ethnicity also contribute to unique aging experiences, with LGBTQ+ older adults of colour navigating compounded marginalisation and often underrepresented in research and services [20]. Acknowledging this complexity is essential to avoid flattening the diverse realities of queer aging and to ensure that theoretical frameworks apply to health systems challenges in ways that are sensitive to these intra‐ and inter‐group differences and the specific configurations of power, identity and care they encompass.

The health systems challenge which we seek to examine through a theoretical lens is ‘queer caregiving’ with older adults. To do this, we employ queer theory—a post‐structuralist critical theory that challenges traditional assumptions about gender and sexuality [21]. The purpose of this paper is to (1) discuss the experiences of LGBTQ+ older adults in the context of an informal caregiving role and how heteronormative and cisnormative systems shape caregiving networks; and (2) describe the applicability and utility of queer theory as a lens to move beyond intersectionality and redesign care frameworks. We begin with an overview of the study of queer gerontology and how it can tie itself to the practice of informal caregiving of older adults via ‘queer caregiving’. We assert the need for theory to address the challenges associated with queer caregiving and propose queer theory as suitable to address this. We then examine literature related to caregiving in queer communities through the lens of queer theory, and lastly propose directions for research, practice, and policy.

### 1.1 Queer Gerontology

Over the past decade, there has been a surge in scholarship on ‘queer gerontology’, a theoretical approach that seeks to interrogate and unsettle normative assumptions about aging and later life [22, 23]. Scholars who enact this ‘queering’ of gerontology draw on insights from post‐structuralist, feminist and queer theories to highlight how later life is shaped by heteronormative and cisnormative expectations or, in other words, norms that privilege certain bodies, identities and relationships while marginalising others [24]. This growing field problematizes the binary constructions of ‘successful’ versus ‘failed’ aging, arguing that standard gerontological narratives often assume stable genders, lifelong heterosexual relationships, able‐bodiedness and cognitively intact minds as the normative path through which older adults achieve well‐being. In so doing, conventional frameworks overlook the experiences of LGBTQ+ older adults whose identities and relational structures do not fit neatly into these scripts, leading to their invisibility or misrepresentation in research, care policies and clinical practice [23].

Central to queer gerontology is the recognition that aging is embedded in social, cultural and political networks that continue to privilege heteronormativity, able‐mindedness and biological kinship ties [22]. As this stream of scholarship expands, there is a growing emphasis on the need to ‘queer’ the social networks and care contexts in which LGBTQ+ older adults live. Yet despite this literature, most queer gerontology studies focus on topics such as identity formation, intimacy and discrimination, with relatively little attention to how queer theory could reshape our understanding of informal caregiving partnerships or caring dyads. This gap is significant because caregiving relationships, whether with partners, friends or families of choice, are a vital axis of support for older adults who may already be structurally marginalised [24, 25]. By embedding queer theory insights into analyses of care partnerships, we can examine how heteronormative assumptions about who cares for whom, what counts as ‘family’, and how caregiving shapes the experiences of LGBTQ+ older adults. Such a perspective foregrounds the creative forms of reciprocity, resilience and kinship building that often exist outside traditional family norms, thus broadening what ‘care’ can look like in later life.

## 2 What Is ‘Queer Caregiving?’

Within the scholarship of queer gerontology fits ‘queer caregiving’ with older adults. Queer caregivers' experiences disrupt traditional heteronormative assumptions about caregiving, which often assume a heterosexual and binary gender framework [26]. They embody diverse caregiving structures that go beyond biological kinship, emphasising their chosen families. These structures can encompass a wide range of factors, including societal attitudes towards LGBTQ+ identities, the availability and accessibility of LGBTQ+‐inclusive healthcare services, legal protections (or lack thereof) for LGBTQ+ individuals, and the representation of LGBTQ+ individuals—particularly older adults—in health research and education. As such, understanding the unique challenges and strengths of LGBTQ+ older adults and their caregivers is vital for effective healthcare practice [27].

Research highlights how normative structures, often based on heteronormative and cis‐normative assumptions, can impact LGBTQ+ health in several ways [25]. The dominance of heteronormativity and cisnormativity in healthcare settings can lead to a lack of culturally competent care for LGBTQ+ individuals, making it challenging for them to access services that meet their specific needs [28]. Normative structures can also contribute to the stigma and discrimination faced by LGBTQ+ individuals, which are significant barriers to accessing healthcare and are associated with poorer health outcomes [29]. Understanding normative structures in LGBTQ+ health and caregiving is crucial for developing inclusive healthcare policies and practices that address the specific needs and challenges of LGBTQ+ populations.

However, due to a lack of research, addressing these needs remains a challenge [30]. The experiences of queer caregivers also reveal the need for development of specialised services and advocacy to eliminate discrimination faced by these populations [31]. Educational gaps among healthcare professionals pose a significant barrier to providing person‐centred care to older adults who identify as LGBTQ+, indicating a need for curriculum development and safer physical and social environments [32]. Addressing the needs of queer caregivers requires a multifaceted approach that includes expanding research evidence, developing inclusive policies and practices, and enhancing educational and support systems for caregivers and healthcare professionals. These dynamics demonstrate that queer caregiving is a set of practices that exist within and are often constrained by heteronormative and cisnormative systems. We must move from documenting and collating queer caregiving experiences [25] to transforming them. This requires a theoretical lens: one that is capable of interrogating power, decentring fixed categories of family and identity, and illuminating the creative care formations that already sustain LGBTQ+ older adults.

### 2.1 The Need for Theory

Queer theory offers this analytic leverage. It highlights the need to recognise and address the marginalisation that queer caregivers experience in healthcare and social support systems. Their experiences often remain invisible in mainstream discussions about caregiving, reflecting a broader societal marginalisation [33]. By treating identities and relationships as fluid, rather than fixed, queer theory aligns with the lived realities of care trajectories common to LGBTQ+ older adults and queer caregivers. Queer theory offers a critical lens for challenging social and health systems that privilege heterosexuality and a binary system of sex and gender by amplifying the non‐normative narratives and experiences of LGBTQ+ older adults and caregivers. It interrogates traditional binaries and emphasises the fluidity of identity and experience [24, 34].

Applied to aging and caregiving, queer theory enables a deconstruction of societal norms and expectations that often marginalise LGBTQ+ older adults and caregivers, providing a deeper understanding of their unique challenges and strengths. For example, LGBTQ+ older adults and their caregivers frequently encounter quality of life challenges such as discrimination, anticipated discrimination in social and healthcare settings, and lack of family support [35]. Concurrently, they demonstrate resilience, reflected in their capacity to adapt to adversity and develop innovative support networks that challenge heteronormative models [36]. These findings, drawn from mainly qualitative research, emphasise the need for social and healthcare that is sensitive to these unique strengths and challenges [35].

The distinct life experiences of LGBTQ+ older adults affect their ability to receive adequate care, highlighting the need for inclusive services and caregiver support [37]. Consistent with queer theory's emphasis on fluidity, queer caregivers often navigate flexible and non‐traditional roles in heteronormative health systems [38]. Their caregiving responsibilities might shift and evolve, challenging rigid definitions of what caregiving entails. Moreover, queer theory's intersectional approach is pertinent, as queer caregivers may face multiple layers of marginalisation (e.g., based on race, age, socioeconomic status, sexual orientation, and gender identity). Understanding these intersecting identities is crucial for comprehensively addressing their needs.

## 3 Overview of Queer Theory

Queer theory challenges conventional categories of identity and cultural views of normalcy. Its fundamental components include a critique of normativity, the fluidity of identity, and resistance to categorisation. Queer theory interrogates the binary of heterosexuality and homosexuality, arguing for a broader spectrum of sexual identities, including bisexuality [39]. This perspective sees identity not as a fixed attribute but as fluid and continually in flux. Hall [21] emphasises the diverse and complex nature of queer theory and its intersections with other critical theories and social issues: sexual identity is a socially constructed concept, which has gained significant political and social implications, particularly in the late nineteenth century.

Hall [21] further describes the interconnectedness of sexuality with social existence, highlighting how various forms of sexual expression are legitimised or condemned based on religious, economic, and cultural paradigms. Furthermore, categorisation and binary definitions often oversimplify complex human experiences [21]. Drawing upon insights from post‐structuralism and post‐modernism, as well as philosophers like Butler [40], Foucault [41] and Sedgwick [42], a queer analysis of sexuality that challenges normative constructs can be articulated. While queer theory can enrich understanding, it must also balance a recognition of the social and institutional realities shaping sexual identities and practices [43].

Queer theory operates on several foundational beliefs, such as the social construction of sexual identities and the challenge to binary thinking [44]. It problematizes and destabilises all sexual identities and practices, inviting a questioning of all sexualities and a critical unpacking of normative categories. It seeks to reveal the queerness of normalcy and the conventional definition of what is considered ‘queer’ [44]. There is an emphasis on including a wide range of sexualities in discussions, moving beyond limited identity categories such as LGBT to encompass a broader range of sexual practices and gender identities (i.e., Q+ in LGBTQ+). This approach challenges the binary understanding of hetero/queer identities [44]. Regarding aging, queer theory can address issues such as isolation, health behaviours, mental health, and the impact of marginalisation on LGBTQ+ older adults, highlighting the need for more inclusive approaches in healthcare and caregiving [45]. As well, there is a call for more integrated approaches to understanding sexual and gender minority experiences across the lifespan, addressing gaps in research on LGBTQ+ families [46].

Queer theory emphasises the fluidity of identities and resists binary categorisations, advocating for a more inclusive understanding of family structures beyond biological or legal ties [47]. This perspective is particularly relevant in the provision of family‐centred care, where queer theory encourages the questioning and dismantling of cisgender norms, thus promoting a more flexible approach to individual and relational identities [47]. The concept of ‘chosen family’ among LGBTQ+ individuals highlights the significance of non‐biological kinship structures in providing mutual care and support, especially in the context of health and illness [48]. The concept of a chosen family reinforces the idea that informal caregiving in queer communities often transcends traditional definitions, fostering resilience against societal discrimination and exclusion [48]. However, queer theory also prompts us to consider the limitations and challenges that remain unaddressed in family caregiving, particularly the experiences of discrimination, marginalisation, and the need for more inclusive healthcare practices. Brotman et al. [31] shared that caregivers of gay and lesbian older adults encounter unique challenges, including discrimination and a lack of recognition, which can impact the quality of care and support available to them.

### 3.1 Criticisms and Intersectionality

Despite its strengths, queer theory faces criticisms. Brown [49] addresses the theory's complexity and potential for over‐generalisation. One critique is that its abstract nature may hinder its applicability in practical settings, posing challenges for those seeking to use its insights in fields like social work, healthcare and education [49]. Another concern is that its broad scope can sometimes lead to an oversimplification of the diverse experiences within the LGBTQ+ community [49]. Intersectionality, on the other hand, is a framework for understanding how multiple social identities and systems of power interact to shape unique experiences of privilege and oppression [50]. However, queer theory uniquely destabilises identity categories and normativity in ways that intersectionality cannot on its own [51]. Queer theory not only interrogates social norms but also challenges the normative assumptions embedded in research and practice paradigms themselves, including those within intersectionality [51, 52]. Lastly, where intersectionality often seeks coalition building among marginalised groups based on shared or intersecting identities, queer theory emphasises an anti‐categorical approach and resists assimilation into existing social structures [51, 52], such as that of family caregiving.

## 4 Queer Caregiving in LGBTQ+ Communities

To redefine informal care partnerships and caregiving dyads for LGBTQ+ older adults beyond traditional heterosexual frameworks, we now draw from queer theory and empirical research that recognises the diverse, non‐normative caregiving structures within LGBTQ+ communities. This approach not only broadens our understanding of who can be a family caregiver but also challenges normative assumptions about care, kinship and relationality.

### 4.1 Navigating Heteronormative Health Systems

Consistent with queer theory's focus on intersectionality, Fredriksen‐Goldsen et al. [53] emphasise the need to understand LGBTQ+ aging through multiple intersecting identities, such as race, class, gender identity and sexual orientation. This acknowledges the complex and varied experiences within the LGBTQ+ community, moving beyond a ‘one size fits all’ understanding of aging. Fredriksen‐Goldsen et al. [53] use queer theory to critique and move beyond heteronormative assumptions in aging research. Heteronormativity, the assumption that heterosexual relationships are the default or norm, has traditionally influenced research on aging [53]. Heteronormativity in aging can negatively affect health outcomes for LGBTQ+ older adults, particularly in the context of caregiving, through various systemic, institutional and interpersonal channels. For example, staff in residential aged care facilities often operate under heteronormative assumptions, where heterosexual orientation is implicitly considered the norm [54]. This can lead to heterosexism, or prejudice against LGBTQ+ individuals, affecting the inclusivity and sensitivity of the care provided [54]. Another example is the experience of older women who identify as lesbian. Their experiences, reported in a study by McIntyre and McDonald [55], highlight the tensions between the rights of LGBTQ+ people and heteronormative healthcare practice. Anticipatory dread of the erasure of their lives and identities in residential or long term care underscores the need for healthcare institutions to move beyond heteronormative ideals [55]. By focusing on LGBTQ+ experiences, Fredriksen‐Goldsen et al. [53] challenge these norms and provide a more inclusive understanding of aging.

Queer theory encourages us to question normative assumptions about who should provide care and how care should be provided, potentially leading to more inclusive and flexible caregiving arrangements that accommodate the diverse needs and capabilities of caregivers and care recipients. While there is a paucity of current literature that directly addresses queer theory in the context of caregiving, other research offers insights into the complexity of caregiving relationships and the importance of addressing relational dynamics, satisfaction, and the need for support in caregiving contexts [56, 57, 58]. Recognising and addressing power imbalances within caregiving relationships is critical [58]. Power dynamics can affect the distribution of caregiving responsibilities, access to resources, and the ability of caregivers and care recipients to express their needs and preferences [58]. By examining how power relations shape caregiving practices, queer theory can guide the creation of more equitable caregiving environments where the rights, dignity and autonomy of all parties are upheld. Encouraging open, honest, and inclusive communication about needs, expectations and challenges within caregiving relationships can help address power imbalances and improve caregiving satisfaction for both queer caregivers and their care recipients [25, 56, 57].

### 4.2 Challenges and Coping Strategies

The unique challenges faced by queer caregivers of older adults, including issues of discrimination, invisibility and lack of tailored services, are significant and multifaceted [45]. LGBTQ+ older adults often rely on friends for informal care [10]. LGBTQ+ older adult caregivers encounter barriers in obtaining health and aging services, which leads to reliance on friends due to discrimination in health service and legal systems [10]. Rather than relying on relatives, LGBTQ+ older adults largely care for each other, reflecting the importance of ‘chosen family’ and the high level of non‐relative caregiving within LGBTQ+ communities [59]. These caregiving relationships differ from the general population, impacting caregiving demands and resources [10], accompanied by unique challenges navigating heteronormative health systems [25]. For example, LGBTQ+ persons living with dementia often encounter unique challenges in accessing care and support compared to their non‐LGBTQ+ counterparts due to elements of discrimination [24]. This compounds with higher likelihoods of being single, living alone and having no children. Compared to the general population, LGBTQ+ older adults are less likely to have traditional sources of caregiver support and more likely to be acting as caregivers to non‐kin [25, 60].

Studies have begun exploring the needs of queer caregivers and persons living with dementia, revealing significant caregiving burden and mental health issues among queer caregivers [25, 61]. Furthermore, a study focused on older LGBTQ+ women highlighted the fear of future challenges and discrimination [11]. Key themes included the need for healthcare professionals who are supportive and knowledgeable about LGBTQ+ issues, consistent recognition of same‐sex partners as primary caregivers, increased sensitivity training, and more open environments where LGBTQ+ patients and caregivers feel comfortable [11]. It is imperative to recognise and address the challenges queer caregivers face to provide adequate support and care for both the caregivers and the older adults they care for.

## 5 Implications for Research, Practice, and Policy

Queer theory addresses the marginalisation and oppression of those who do not conform to societal norms, particularly in relation to sexuality and gender expression [49] and advocates for the deconstruction of categories, arguing that identity is not fixed but rather fluid and continually being negotiated [21]. Addressing challenges related to queer caregiving in research, practice and policy requires a comprehensive, culturally competent and theoretically informed approach that recognises the specific needs of this diverse community [62].

### 5.1 Future Research Recommendations

While the literature diversely describes experiences of family caregivers [25], wider research priorities should move towards exploring the nuances and specific experiences of caregivers within marginalised populations [13]. For future research, it is imperative to further explore the unique experiences of queer caregivers, with a focus on the compounded challenges faced by those with multiple marginalised identities, such as LGBTQ+ individuals marginalised by race and ethnicity. Studies should aim to uncover the nuanced needs and experiences of these caregivers, informing the development of tailored support services. Additionally, future research should evaluate the effectiveness of queer‐theory‐informed interventions in healthcare and family caregiving practices, assessing their impact on both caregivers and care recipients.

### 5.2 Towards Inclusive and Empowering Practice

Queer theory in relation to queer caregiving involves fostering inclusive and sensitive approaches, especially for marginalised groups such as the LGBTQ+ community and racial minorities. The method of ‘queer phenomenology’ [63] offers insights into how spaces and orientations can be ‘queered’ to resist normative and capitalist structures. The method, offered by Ahmed [63], can be applied to caregiving environments in ways that suggest creating spaces that challenge conventional orientations in both physical and ideological facets [64]. Through these spaces, we can create a more inclusive and supportive atmosphere for those often marginalised by traditional health systems, including LGBTQ+ older adults [63, 64]. Culturally competent care, when informed by queer theory, emphasises challenging heteronormative assumptions and addressing power dynamics in healthcare. It moves beyond ‘learning about’ to ‘learning with’—engaging queer caregivers with lived experiences and recognising the diversity within LGBTQ+ communities, being attentive to how power and norms shape healthcare interactions [65]. Encouraging healthcare professional self‐reflection on power can lead to awareness of biases and systemic inequalities [65].

Hughes [66] emphasises the importance of privacy in aged care, particularly for LGBTQ+ older adults, discussing how privacy practices can both facilitate and limit the expression of sexual identity in these settings [66]. This highlights the need for healthcare professionals to construct environments that enable LGBTQ+ older adults to disclose and express their sexual identity, thus avoiding institutionalised homophobia [66]. However, the application of queer theory extends beyond sexual orientations and gender identities. It offers a framework for understanding and supporting patients and caregivers with intersecting marginalised identities, such as those who are disabled or ethnic minorities [67]. Queer theory promotes inquiry over inclusion, especially in educational contexts. This approach can be applied to caregiving training programmes to explore how sexual identities are negotiated in social interactions, leading to more inclusive healthcare practices [68].

Kessler [69] describes family caregiving as a potential form of political resistance or expression, particularly for people traditionally denied family privacy by the state. This includes ethnic and racial minorities, gays and lesbians, and heterosexual men [69]. Kessler [69] argues that caregiving can constitute an affirmative political practice of resistance against discriminatory institutions and ideologies, including patriarchy, racism and homophobia. The potential of queer theory to inform caregiving lies in its ability to recognise and address the unique experiences and needs of marginalised groups. By acknowledging the intersection of various identities and the historical context of oppression, healthcare professionals can develop more inclusive and sensitive approaches that respect and affirm the diverse identities of their patients and their patients' caregivers. This notion includes creating environments that allow for the expression of sexual identity and recognising caregiving as a form of resistance and empowerment for minority groups.

### 5.3 Policy for Systemic Change

Lastly, regarding policy, there is a pressing need for systemic changes that reflect the principles of queer theory. Policies should strive to dismantle heteronormative assumptions in health systems, ensuring the recognition and support of queer caregivers and their care recipients. This includes implementing training on inclusivity for health and social care professionals, reinforcing non‐discriminatory policies, and ensuring access to LGBTQ+‐sensitive resources and support networks. Ultimately, the application of queer theory in healthcare policy can lead to significant improvements in the quality of care for LGBTQ+ individuals. By challenging heteronormative assumptions and practices, healthcare providers can create more inclusive environments that cater to the specific needs of LGBTQ+ patients, ultimately leading to better health outcomes and patient satisfaction [70].

## 6 Conclusion

Queer theory's emphasis on fluidity, intersectionality, and the deconstruction of normative structures provides a valuable framework for understanding the diverse caregiving arrangements within the LGBTQ+ community, challenging traditional caregiving narratives and advocating for inclusivity. The importance of recognising queer theory in the context of queer caregiving cannot be overstated. It not only brings to light the marginalisation and invisibility these caregivers often face but also emphasises the resilience and adaptability inherent in their caregiving roles. By acknowledging the complexities of their experiences, we can foster a more inclusive and supportive environment for queer caregivers and the older adults they care for [25]. The call for health systems to be more inclusive and culturally competent aligns with queer theory's advocacy for systemic change to accommodate diverse identities and experiences. Through the lens of queer theory, the experiences of queer caregivers can be understood as part of a broader struggle against normative structures and the marginalisation of non‐conforming identities. This perspective underscores the importance of recognising the diverse and fluid nature of caregiving in LGBTQ+ communities and the need for systemic changes that respect and accommodate this diversity [26]. Queer theory thus provides a critical framework for understanding and addressing the unique challenges faced by queer caregivers of older adults. Embracing queer theory as a guiding framework in research, practice and policy will enhance our understanding of queer caregiving and foster a more equitable and compassionate society that values and supports all caregivers, regardless of their sexual orientation or gender identity.

## Author Contributions

**Steven Hall:** conceptualisation, writing drafts and approving final manuscript. **Austin Oswald:** writing and reviewing drafts and approving final manuscript.

## Funding

The authors have nothing to report.

## Ethics Statement

The authors have nothing to report.

## Consent

The authors have nothing to report.

## Conflicts of Interest

The authors declare no conflicts of interest.

## Data Availability

The authors have nothing to report.
